# Supplementary material for: Identification of photoperiod-induced specific miRNAs in the adrenal glands of Sunite sheep (Ovis aries)
Source: Front Vet Sci. 2022 Jul 22;9:888207. doi: 10.3389/fvets.2022.888207 (PMC9354845; doi:10.3389/fvets.2022.888207)
Supplement: Supplementary file 1 [file Data_Sheet_1.ZIP › Supplementary file/miRDeep2 file.zip!/miRDeep2 file/Novel_Structure/LP42A3/Novel_413.pdf]

Provisional ID	:	NC_019459.2_3122
Score total	:	2
Score for star read(s)	:	-1.3
Score for read counts	:	0
Score for mfe	:	1.7
Score for randfold	:	1.6
Score for cons. seed	:	
Total read count	:	61
Mature read count	:	60
Loop read count	:	0
Star read count	:	1

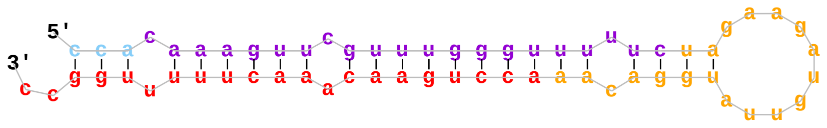

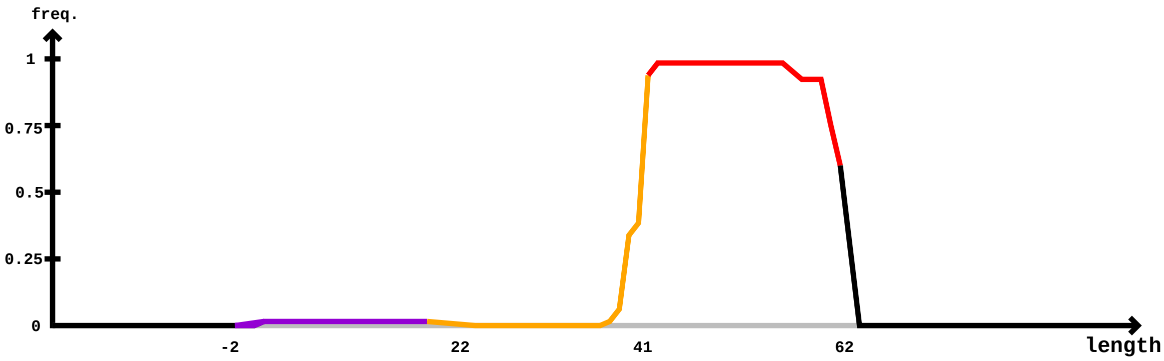

Star **Mature**

```
5'- auaugguauuaaauuggccacaaaguucguuuggguuuuucuagaagauguuauggacaaaccugaacaaacuuuuuggccagcccaauaccauuaggugccaucuacuaa  -3'  obs
    auaugguauuaaauuggccacaaaguucguuuggguuuuucuagaagauguuauggacaaaccugaacaaacuuuuuggccagcccaauaccauuaggugccaucuacuaa        exp
    ..((((((((...(((((((.((((((.((((((((((.((((..........)))).)))))))))).)))))).)))))))...))))))))(((((...)))))....   reads    mm    sample
    ....................caaaguucguuuggguuuuuc......................................................................   1        0     seq
    ........................................................aAaaaccugaacaaacuuuu...................................   1        1     seq
    .........................................................Aaaaccugaacaaacuuu....................................   2        1     seq
    .........................................................Aaaaccugaacaaacuuuu...................................   1        1     seq
    ..........................................................aaaUcugaacaaacuuuuugg................................   11       1     seq
    ..........................................................aaaccugaacaaacuuuuuggc...............................   3        0     seq
    ..........................................................aaaAcugaacaaacuuuuuggc...............................   1        1     seq
    ..........................................................aaaccugaacaaacuuuuuggU...............................   3        1     seq
    ...........................................................aaUcugaacaaacuuuuuggc...............................   1        1     seq
    ...........................................................aaUcugaacaaacuuuuuggcc..............................   2        1     seq
    ............................................................aAcugaacaaacuuuuuggc...............................   1        1     seq
    ............................................................accugaacaaacuuuuuggcc..............................   1        0     seq
    ............................................................aUcugaacaaacuuuuuggcc..............................   34       1     seq
    .............................................................Ucugaacaaacuuuuuggc...............................   1        1     seq
    .............................................................Ucugaacaaacuuuuuggcca.............................   2        1     seq
```
